# The Treatment of Pulmonary Consumption

**Published:** 1894-08

**Authors:** Allen J. Smith

**Affiliations:** Professor of Pathology and Lecturer on Mental and Nervous Diseases in the Medical Department of the University of Texas, Galveston


### TEXAS MEDICAL JOURNAL.

ESTABLISHED JULY, 1885.

PUBLISHED MONTHLY.—SUBSCRIPTION $2.00 A YEAR.

VOL. X. AUSTIN, AUGUST, 1894. NO. 2.

### Original Contributions.

For Texas Medical Journal.

### THE TREATMENT OF PULMONARY CONSUMPTION.

BY ALLEN J. SMITH, M. D.,

Professor of Pathology and Lecturer on Mental and Nervous Diseases in the Medical Department of the University of Texas, Galveston.

[Read before the Galveston County Medical Society, January 28, 1894, and published by order of the Society.]

THE almost uniform failure by the profession to cure cases of pulmonary consumption has become nearly proverbial, the stigma of incapacity being accepted with scarcely an attempt to excuse or explain, in no matter what stage the disease may present itself; and the recognition by the physician of positive signs of the affection has almost come to be tantamount to his mental signature of the patient's death-certificate. Not that there have been no efforts, individual and concerted, toward the establishment of a rational means of cure of the malady,—there have been numerous endeavors, first and last; but almost without exception, they have proved either absolute failures, or have presented such a modicum of success that they have been regarded as practically worthless. Within the past ten or fourteen years, dating from the time of Koch's demonstration of the micro-organismal cause of the disease, the utmost therapeutical activity has been manifested, such earnestness in the pursuit of a remedial measure suitable for the destruction of the tubercle bacilli having perhaps never before been witnessed in the study of the management of disease; yet of all the microbicidal agents and measures proposed, not one has withstood, save in isolated instances of use, the test of a few months or a few years practical employment by the profession at large. The truth of such a confession is all the more forcibly borne upon one if he has had any close relations with any of the various projects for the alleviation and cure of pulmonary cases—the compressed air method, the rarified air method, Bergeon's rectal injections of sulphurretted gas, or the various biological methods, as that of Koch in Germany, or Dixon in this country.

Very recently (*New York Medical Record*, December 18, 1893) there has been published an article from the pen of Dr. Hershey, of Denver, the object of which is to call the attention of the profession to this phase of the present status of our dealing with consumption; the author strongly, and very truthfully, if the writer's experience may be offered as corroborative, insisting upon the practical absence of value in any of the measures having the direct purpose of destroying the tubercle bacilli, and even the occasional detrimental influence of such measures.

It may seem gratuitous, yet at that very risk, after such an introduction, the writer must nevertheless indicate his adherence to the belief and hope for the eventual discovery of just such a bacterial destructive agent through which once and for all the cause may be eliminated from the system, and the disease cured. It is even within the scope of this paper to state his belief in the eventual success of efforts directed along the lines suggested by Koch in Europe and Dixon in America, in the discovery of an agent derived from the bacteria themselves, or procured through their direct agency, from some organic source, which will have the power of destroying the bacteria of consumption without seriously modifying the structures within which they may be existing, or the power of inducing an eliminative force in the economy which may in some way separate the diseased structures permanently from the healthy.

For the present, however, the writer is strongly disposed to agree with Dr. Hershey in his assertions that the prevailing modern therapeusis of phthisis pulmonalis is, as a rule, only a menace to continuance of life and a detriment to the fulfillment of cure. There is far too much disposition to rely on the hoped for and often asserted specific germicidal activity of this or that remedial agent, and too little regard for the ordinary environments of life in their relation to the preservation as well as to the return of health—even more, too little regard for the possible untoward results of the bacteria-destroying agent, lest it also be tissue and function destroying. Within the past few years the indubitable danger of interfering with, if not of destroying the gastric function by such agents as creasote, eucalyptol, oil of cloves, turpentine, alcohol, and a host of similar agencies, has been forced upon the writer's recognition by case after case. It is quite possible that these remedies may, in the bacteriologist's tube, if used in excess, destroy the vitality, or perhaps the specific possibilities of the tubercle bacilli, but in the ordinary case of consumption they have seemed in many instances to the writer to exert as strong a lethal power on the patient's body as upon the bacteria. Their curative power is, it is true, demonstrated in isolated cases where the incipiency of the disease and their proper administration, together with due consideration for hygienic influences, have concurred in offering the most favorable conditions for cure, yet these comparatively few instances of success only serve to more powerfully emphasize their usual failure. The one almost universal fault in the use of these remedies is the failure to properly appreciate their irritative influence on the alimentary tissues, and their ability, in large doses, to subvert the alimentary function. A digestion already impaired by the inefficiency of the digestive juices caused by the hæmic alterations of the pretubercular anæmia, cannot by any possible process of reasoning be expected to recover its integrity by the production of a gastritis, often of decided severity, by the administration, up to and beyond the point of tolerance by the patient's body, of badly diluted irritants, such as oil of cloves or creasote; it must, on the contrary, suffer the more, and the economy, whose condition is dependent as much on the preservation of the nutritive functions as upon its freedom from parasitic irritation, must eventually severely decline.

Realizing these dangers and the almost sure defeat of purpose by adhering to the pharmaceutical means in vogue at present among the greater part of the profession, and keeping well in mind the fact that the principles of hygiene are not altered in disease from those in health, although there may be slight modifications in its practice, the writer has endeavored, in four cases falling to his care within the past year, to lay aside existing methods and to return to a careful observance of the rules of procedure so often suggested by the older practitioners. Dr. Hershey quotes Professor Alonzo Clark to the effect that by observance of proper hygiene, by out-door life and exercise, by forced feeding, together with perhaps some simples occasionally, phthisis is curable.

The writer's observation of these few cases strongly corroborates such a statement; probably had all the conditions in a large number of other phthisical cases, which have from time to time been under care of the writer, been possible, as in these four, the list of favorable results might well have been added to. In two of these cases, the tubercle bacilli had been demonstrated, one of these two having been told by a noted physician of Chicago that he could not live more than six months at the outside. In one other physical signs were easily made out, but the bacilli, for certain reasons, had never been sought for. In the fourth, there had been a number of pulmonary hemorrhages, indistinct signs in a limited area of the chest, but no expectoration save forced, and no bacilli in that. In all these cases marked improvement followed immediately upon the commencement of the line of treatment to be mentioned below. All of these cases have been under observation for some months,—one nearly a year, two more for ten months, and the fourth three or four months. One has gained from 107 to 143 pounds, another from 126 to 154, another had originally weighed 220, dropped before treatment to 140, and gained to 160 at last account, while the fourth gained from 137 to 187. Three of these cases the writer knows to be in seeming good health, apparently as well or better than ever in their lives; one has not been heard from for several months. The report at that time stated that he had caught a severe cold in the sleeping car on his return journey from this State to Chicago, and had been confined to his bed for several days, but that he was again out, and improving. Whatever his present condition, the fact remains that while he was under proper conditions he constantly and rapidly improved. Two of these cases might ordinarily be pronounced cured were it not for the well known danger of such announcements until after protracted periods of time have intervened after the last presentation of active symptoms.

The treatment which the writer regards as that best relied upon, judging from the practice of our elders as well as from our own failures and successes, is one which endeavors to overcome the disease simply by strengthening the patient's general condition, by seeking to give the body power to resist further encroachments and to isolate already diseased areas. Every effort to preserve the integrity and to increase the functional energy of the nutritive organs is to be made. Every effort consistent with such care of the nutrition is to be made to prevent pulmonary hyperæmia, to facilitate pulmonary expansion and to aid the pulmonary lymphatic drainage. Every effort consistent with both these suggestions must be made to overcome the microbic and toxic agents at work at the bottom of the disease. These features of treatment can best be carried out in a dry equable climate, by rest (often absolute), by forced nutrition, by the careful administration of tonic stimulants and stomachics, by proper pulmonary gymnastics, and by the judicious use of alcohol.

In the matter of climate, the trend of professional opinion has wandered from the warm, moist, equable climates to the cool, dry and elevated atmospheres of the mountainous regions. There are dangers in either extreme, and the desideratum is to be found in an atmosphere of the driest possible character, pure and uncharged with particulate elements from dust or manufactures or disease, with only the moderate variability which does not wear upon the system, and with a pressure sufficient not to predispose to pulmonary hyperæmia and consequent hemorrhage. The moister, warmer, equable or changeable climates of the seacoast favor too highly the active development of the bacteria in the lungs, the experience of most practitioners at the seashore amply verifying such a statement; on the other hand, the cool (and necessarily variable), dry and light atmospheres of high elevations too frequently are employed only to have a sudden hemorrhage end the case. It is exceedingly fortunate for the practitioners of this State that within their own commonwealth exists perhaps the nearest approach to the desideratum in the entire country, if not in the known world.

Within a circle, taking in the counties of Kendall, Kerr and Gillespie, and portions of Blanco, Llano, Mason, Kimble, Edwards, Bandera, Bexar, Comal and Hays, there exists a climate than which a better for the purposes under consideration probably does not exist. The extreme range of temperature as in most dry climates is considerable, from 104° F. to as low as 20° F. upon one occasion, as registered at San Antonio*; this range,

*These data were obtained from an unpublished monograph upon the climate of Southwestern Texas, by the late Dr. Morse K. Taylor, Major and Surgeon in U. S. Army.

however, is by no means so marked as many climates present which are regarded as not unfavorable to health or even to the cure of phthisis. For example, at Boston there are not infrequently summers in which a maximum of 100° F. is attained, and it is the rule for the thermometer to register considerably below zero each winter; in Colorado the extreme range of temperature is far beyond that indicated for the San Antonio region, by twenty or thirty degrees at least. The mean annual range for the district mentioned is, however, much less than the extreme, from 98° F. to 28° F., and while the number of days that the thermometer stands above 90° F. may reach to more than 90, the days when the thermometer has fallen to 40°, or below, are rarely more than ten or fifteen. The range obtained by showing the average temperature for the hottest and coldest months, which, therefore, gives the mean extreme range for the year, a still more true indication of the variations to be expected, is not even as marked as the last, the mean temperature for July being 82.9°, and for January 50.6°, a mean extreme range much less than is to be found in any other of the dry climate resorts. The mean daily variations in temperature, from mid-day to mid-night, are about twenty or twenty-four degrees, insuring the coolness necessary for comfortable sleeping; yet this degree of variation leaves the minimum some six or seven degrees above dew-point, and the nights as the days are dry. Such conditions are, moreover, conducive to the spending of a large part of the day and night in the open air, a feature of importance in the care of the phthisical well recognized by all physicians. The prevalence of southern breezes from the gulf renders pleasant the warm temperature of the day, and the fall of temperature at night is gradual, and wraps are not required until sleep is sought. The climate has a distinctly bracing effect, readily felt by persons going from the coast inland to the district named; and, contrasted with other resorts commended for pulmonics, in matter of uniformity (with sufficient variation to render the patient comfortable), of warmth and dryness, there are few points to be found fault with. In this district the elevation varies from about six or seven hundred feet above the sea level to sixteen or seventeen hundred feet, an elevation and openness of country sufficient to assure one of the purity of the air, yet having sufficient pressure to prevent predisposition toward hemorrhage. Some portions of the district are rendered unsuitable from the prevalence of alkaline dust, often as dust storms, but these may be readily avoided by choice of locality. My own preference in location of patients in this district is in the country around San Antonio, where the comforts of a city may be obtained in a short while and at reasonable expense, and where freedom from the heat and dust and noise may also be found.

The question of nutrition next demands attention. It should be ample and even forced. A glass of warm milk, or a weak milk punch, is well borne by most stomachs soon after the patient awakes in the morning, and is quite sufficient to overcome the tendency to cough in the incipient stage of the disease. The three full meals a day, with a large proportion of animal food, urged, if necessary, upon the patient, varied frequently to persuade the appetite, should be insisted upon; and the utmost importance attached to slow eating and full mastication and salivary admixture. At meals a decided stimulant to the appetite, an aid to digestion and a valuable nutrient is afforded by brown stout or porter—far preferable to wines or ordinary beers. Its bitter taste, if objectionable to the patient, may be rendered pleasant by sugar and a bit of nutmeg. Between meals, in the morning, afternoon, and evening if unfortunately late hours be kept, a little alcohol, in the shape of milk punches, iced if desired, serves an excellent purpose. Alcohol should always, in the treatment of phthisis, be regarded as one of the most valued and trustworthy remedies; but it is questionable whether whisky, or any of the strong alcoholics, should ever be used save for extraordinary purposes, in an undiluted form, the best dilution being with milk. Alone, whisky is too irritant to the gastric mucous membrane, and loses much of its value by finally overcoming the stomach and its power for work. Besides its action as a stimulant and nutrient, alcohol has, in more than the cases cited, seemed to the author to possess a decided value in antagonizing the toxic products of the micro-organisms of the disease. There are many who decry its value and loudly assert its impotency and detrimental power, but the writer is willing to join such only to the extent of condemning the stronger alcoholics in their excessive use and undiluted form. In addition to the porter and whisky thus disposed of, there is no reason why a short time before meals there should not be taken, at least occasionally, some good bitter tonic as nux vomica, or some of its preparations with the hypophosphites. This, however, by no means completes the scheme for nutrition. Aside from the table, nothing is more to be insisted upon than the use of cod-liver oil—not taken in infinitesimal doses at long intervals or in the nondescript conglomerations of many of the pharmaceutical preparations being constantly foisted on the profession—but as plain cod-liver oil. Of all the oils with which the writer has had any experience, none has so well suited his patients' tastes as that of Möller, an oil claimed by the manfacturer to be pressed by a cold process from daily fresh livers; it is a very fluid oil, and relatively not a bad-tasting one. The oil had best be given at least twenty minutes or half an hour after meals, inasmuch as its digestion is accomplished in the intestine; and one may expect by that time that the food is being passed into the intestine. Administration with or before meals can do no special good, and is likely to bother the peptic digestion perhaps, and may lead to eructations tasting of the oil and the consequent development of an aversion for the remedy. If necessary, at first the oil may be administered upon a few drops of whisky placed in the spoon, but this had best be gradually given up and the full dose of the oil (a tablespoonful) insisted upon. Whatever other plans of nutrition may be urged by those opposed to the general principles, the use of the oil as freely as it may be borne is too well established to require further discussion.

In addition to these attempts at nutrition there comes into consideration the question of exercise, general and pulmonary, as necessary to any successful system of caretaking. Alonzo Clark would advise an incipient pulmonic to ride twenty-five miles on horse-back daily. The principle is entirely right, the precipitous practice, as a rule, perhaps injudicious. There is scarcely any exercise which is so well suited to the needs of these cases as horseback riding; it should frequently be practised and gradually increased until Dr. Clark's limits be but play. At first the man who seeks to get pleasure from this measure should not ride at speed, the best gait being the jog-trot so common to Texas horses. The violence of a rough canter or gallop, and the excitement increase the tendency to pulmonary hyperæmia and hemorrhage, and can be approached only after long preparation, and gradually with increasing strength. Other forms of exercise are of course also valuable, but no one who may be in position to enjoy outdoor life and exercise should be permitted to substitute for these any house gymnastics. Walking in the air is far better than the bar within walls. Exercise should always be graded to the capabilities of the system, not up to the limit of absolute tiredness; and frequent and full opportunities should be afforded for rest.

After a period of exercise the patient should not simply sit down to rest, but if opportunity affords he should lie down, and if possible sleep, having divested himself of the clothing dampened from perspiration, and rubbed himself thoroughly dry with a coarse towel. This habit of friction of the surface of the body, both after exercise and at least twice daily, morning and evening, even if no exercise has been taken, should be made an important feature. Special caution should be given that under no consideration is the patient after becoming overheated by exercise to sit in a draught of air that he may become cool; draughts are to be avoided entirely, especially when the individual is inactive. It is often an excellent plan to have the patient with regularity each week choose a day for absolute rest in bed, such a rest having decided value in preserving strength and in aiding in making good tissue loss. Alone, or with forced feeding, and without the valuable aid of climatic and hygienic surroundings, brief periods of absolute rest in bed have proved to the writer in a number of cases to have decidedly beneficial effects, leading in several instances to almost immediate increase in weight and improvement in the general condition. Not only are physical rest and exercise to be sought; but as well, and in many cases even more, mental rest from business worry and from fretting over the physical condition and from fear of death, with exercise of the mind in pleasant directions. This mental rest should be sought in every way, and it is not to be forgotten that oftenest it comes only with suitable bodily occupation.

Quite as important, and in some respects the purpose of the physical exercise, is proper exercise for the lungs. Pulmonary gymnastics in early cases are to be most warmly advised, their practice being limited only when there is danger of hemorrhage. Thus, in cases whose first symptom has been a hemorrhage more or less marked, it is advisable that the patients do not expand the lungs to the fullest extent until the tendency to hemorrhage has disappeared for several months at least. In beginning systematic exercise of the lungs the patient should be instructed to take at intervals of an hour or thereabouts during the day several full breaths, and to endeavor to practise slow deep breathing for a few minutes at a time. This should be done wherever the patient may happen to be, best in the open air as when walking, riding or driving. It is to be urged too that the erect posture be assumed, shoulders thrown back and chest forward, the abdominal muscles being at the same time held voluntarily from movement or protrusion. Thus gradually the tendency to diaphragmatic breathing is lessened, the expansion of the upper portions of the chest increased, aeration of the upper parts of the lungs and consequent increase in their drainage and vascularity brought about. It is to be advised that all efforts toward increase of chest room be practised, with the full expansion of the pulmonary air cells thus afforded. Movements of the arms, synchronous with deep, full inspirations and expirations, as one would move the arms in slow dumb-bell exercise, should be advised. It is a good plan too that the patient should be made to stand in the corner of a room, face toward the corner, and with the body supported against the wall by the outstretched arms, should then with slow deep inspiratory and expiratory movements bend towards the corner and recover the original position. Another excellent movement is to stand near some immovable object so that with outstretched arm the finger tips barely touch the object, then move away an inch or more and without inclining the body endeavor to extend the hand so as to touch the object. This should be done several times and then the opposite side exercised in the same manner. Marked increase in breadth of shoulders and fullness of chest will follow in a few weeks conscientious practice of these simple rules.

The clothing of the patient is another point upon which the physician should bestow his attention; and no better general rule may be laid down than that every phthisical patient should be required to wear woolen garments, at least woolen undergarments. The question of expense and the objectionable feature of woolen fabrics to shrink are often to be considered in the advice to patients who cannot afford to spend much money, but these should not be allowed to weigh heavily where the garments may be had legitimately. By care in washing, shrinkage is much lessened, and if the body surface be kept scrupulously clean, simple airing of the garment in the sunshine will so freshen it, that washing more than once in two weeks is superfluous. The clothes should be often changed and thoroughly aired and dried before being again put on; the body rubbed well from head to foot on changing garments with a towel to promote the surface circulation and to prevent catching cold. If this practice of rubbing the surface from head to toe be carefully carried out several times each day, a weekly bath in winter or a semi-weekly bath in summer will be quite sufficient for purposes of cleanliness and may be practised without danger, the temperature of the bath and the room being carefully looked after. After a bath it is a good plan to lie down for a few minutes until the gentle fatigue of the bath has worn off. Sponge baths where from some reason the full bath may not be indulged in are quite sufficient; and it is well after bathing to annoint the surface with some bland oil and rub it well in, following it with a good toweling until the skin glows. These suggestions as to the bath are of course intended for the incipient stage and are to be modified where the strength of the patient demands.

In the treatment of this disease in its full course there are many complications to be expected in the various stages, and many symptoms which become so important as to require special attention—the cough, the profuse and weakening night-sweats, hemorrhage, the loss of appetite, or the appearance of a tubercular diarrhœa. These, however, must be passed over without consideration, because of the already too great length of the present communication; and the writer must content himself to conclude after a brief consideration of the disposition of the sputum and the infectious nature of the affection. This latter feature is now recognized widely, and one no longer needs defend a belief in the contagiousness of the disease. It is certain, too, that close association, sleeping in the same room and bed, eating and drinking from the same dishes, kissing, and the many other associations of intimate life with the tubercular, are conducive to the spread of tuberculosis. The contagion is often, perhaps in most cases, escaped because of the strength of the unaffected; and this avoidance of infection is easily secured by care. The writer is confident that by far the greatest danger lies in the presence of dried tubercular sputum in the atmosphere inhaled, and believes that by taking especial care in this direction the greatest source of the spread of the disease would be averted; and if, besides this, the common and decent rules of cleanliness be observed, the danger would be of a minimum. For example, the tubercular should not sleep with the non-tubercular; should be instructed not to expectorate promiscuously about habitations, or street cars, or other places where persons are apt to congregate; should carefully rinse the mouth after expectoration, and should use glasses and table instruments that are not in general use. Such precautions would go a long way toward lowering the present frightful morbility of tuberculosis. The sputum should be expectorated upon old cloths and then burned; other disposition is incomplete in comparison to this. By the distribution of information upon these points, and by calling public attention seriously to their consideration, the Pennsylvania Society for the Suppression of Tuberculosis, of Philadelphia, has, in the five years of its existence, accomplished a lowering by 25 per cent. of the tubercular death rate of that city; and if the same good results be obtained each year, it would naturally lead to the practical stamping out of the disease in the next fifteen years. This is, of course, an optimistic view, but there is no doubt, from the brilliant results already gained, that a lowering of the mortality by consumption to one-half the original rate will be accomplished. Texas, with her magnificent climate, so well suited to the need of these unfortunates, will naturally become the point of concentration of tubercular individuals, and the need of concerted effort in this direction must be perfectly apparent, lest in the future, when the advantages of the climate become known, residence in the district above referred to become not a benefit as now, but a menace toward infection. The author proposes, therefore, to presently urge this matter upon the attention of the profession with a view of organization after the manner of the Philadelphia society, and would be glad to enter into correspondence with others having the same subject at heart and the same way of looking at it.

